# Integrating Choosing Wisely, Value-Based Care Principles, Into Undergraduate Medical Education: A Pilot Study

**DOI:** 10.7759/cureus.56912

**Published:** 2024-03-25

**Authors:** Omar El Fadel, Zachary N Goldberg, Amiti Jain, Nitya Venkat, Anjali Upadhyaya, Shale Mack, Mitchell Kaminski, Dimitrios Papanagnou, Deborah Ziring, Geoffrey Hayden

**Affiliations:** 1 Internal Medicine, Thomas Jefferson University, Philadelphia, USA; 2 Family and Community Medicine, Thomas Jefferson University, Philadelphia, USA; 3 Emergency Medicine, Thomas Jefferson University, Philadelphia, USA

**Keywords:** health systems science, medical education, value-based care, resource stewardship, high-value care, choosing wisely

## Abstract

Background: Healthcare spending represents a large portion of the GDP of the United States. Value-based care (VBC) seeks to decrease waste in health care spending, yet this concept is insufficiently taught to medical students. The Choosing Wisely Students and Trainees Advocating for Resource Stewardship (STARS) campaign promotes initiatives that integrate knowledge of VBC into undergraduate medical education (UME). This study sought to determine the most effective strategy to educate medical students on key principles of VBC as taught by the STARS campaign.

Methods: Choosing Wisely principles were incorporated into the UME curriculum of an academic medical institution via the creation of eight new learning objectives (LOs) for case-based learning (CBL) sessions and lectures. Medical students completed an annual 10-question survey from 2019 to 2022 and 10 formal examination questions during the preclinical (years 1 and 2) curriculum after exposure to varying quantities of LOs. Pearson correlation, chi-square, and logistic regression were employed to determine the association between increased LOs in the curriculum and (1) campaign awareness and (2) knowledge of VBC principles.

Results: A total of 700 survey responses over a four-year period (2019 to 2022) were analyzed. Student awareness of the campaign and knowledge of VBC principles increased year over year during the survey period (39% to 92% and 64% to 74%, respectively). There were significant associations between increased LOs in the curriculum and (1) campaign awareness (0.828, p<0.0001) and (2) knowledge of VBC principles (0.934, p<0.001). Students also performed well on formal examination questions related to VBC principles (mean: 81.5% and mean discrimination index: 0.18).

Conclusion: Integration of VBC-focused LOs is significantly associated with awareness of the Choosing Wisely STARS campaign and knowledge of VBC principles taught by the campaign. Collaborative initiatives to increase exposure to VBC education may improve students' knowledge of these principles during medical school.

## Introduction

Healthcare spending remains a major portion of the United States’ gross domestic product. It is forecasted to grow by 5.5% annually and comprise 20% of the economy by 2030. However, 30% of this spending is estimated to be wasteful and does not improve outcomes or benefit patients [1-3]. This excess spending is attributed to failures of care delivery, mismanaged care coordination, and over-treatment, producing nearly $204 billion of estimated waste and exacerbating the affordability crisis that plagues our system [2]. Value-based care (VBC) seeks to decrease waste in health care spending, yet this concept is insufficiently taught to medical students. It is unclear what the most effective strategy is to educate medical students on key principles of VBC.

The Choosing Wisely campaign was introduced in 2012 by the American Board of Internal Medicine Foundation (ABIM), along with consumer reports, to combat physician utilization of low-value care [1-4]. Over 80 associations of various medical specialties have participated in the campaign to produce evidence-based clinical guidelines and draw attention to wasteful services in their respective fields [5]. This includes highlighting tests, treatments, and procedures that clinicians and patients alike should better scrutinize before utilizing. Broadly, the campaign seeks to alter the long-held belief more care is superior to the right care [6].

There is a need to better incorporate high-value care delivery principles early in medical training, such as minimizing waste and medical errors, containing costs, and adhering to established quality metrics. Recognizing this need, the Dell Medical School at The University of Texas at Austin replicated a successful Canadian model and launched the American Students and Trainees Advocating for Resource Stewardship (STARS) program in 2017 [7]. The program culminates in an annual leadership summit emphasizing delegate education, connections between medical schools for future collaboration, and brainstorming sessions to design additional interventions. 59% of the 25 participating medical schools in the United States implemented curricular changes within six months of student representatives attending the first summit and 84% had clear plans to implement changes within one year [7]. Knowledge of these principles fosters greater stewardship and optimized resource utilization in the next generation of physicians.

Traditionally, topics targeting overuse within the healthcare system and redundancy of low-value care are not well covered in undergraduate medical education (UME) [7]. In fact, certain components of medical training may either encourage or discourage a culture of overuse [8,9]. This finding is consistent with the literature demonstrating that physicians trained in environments fostering high-value care demonstrate greater stewardship and optimized resource utilization [10]. To date, few studies have examined the effectiveness of the impact of educational initiatives aimed at increasing awareness and knowledge about VBC [11,12]. This study aimed to determine if longitudinal curricular changes at an allopathic medical school, inspired by the Choosing Wisely campaign, would increase (1) student knowledge of VBC principles and (2) student awareness of the campaign, creating future VBC clinical champions.

We hypothesized that offering students the opportunity to discuss Choosing Wisely recommendations will increase awareness of the campaign and knowledge of VBC principles. This may also shift student opinion toward a greater appreciation for resource stewardship and healthcare costs. This study will serve as proof of principle with the hope that future similar initiatives will graduate clinical champions properly equipped with the necessary knowledge and attitudes to combat waste in medicine and advocate for patients' financial health.

## Materials and methods

Participant selection 

First through fourth-year medical students enrolled at any time between Fall 2019 and Fall 2022 (four academic years) at a single large, private allopathic medical school in the Northeastern United States were eligible for participation in the study. Students all participated in the medical school’s twice-weekly small group case-based learning (CBL) sessions during their first and second years of medical school, which was a mandatory component of the preclinical medical curriculum. These CBL sessions focused on a specific diagnosis and treatment plan related to each systems-based block being taught over the course of the two-year preclinical curriculum. At the conclusion of each CBL case, faculty-derived learning objectives (LOs) were distributed to the students.

Intervention

STARS faculty mentors and medical students collaboratively reviewed the Choosing Wisely recommendations to explore opportunities to integrate important VBC principles into several CBL sessions over the course of the preclinical curriculum. LOs based on the Choosing Wisely recommendations were identified and integrated into existing CBL cases to facilitate discussions regarding value-based health care related to the diagnosis and management of specific conditions discussed during sessions. In total, three Choosing Wisely-focused LOs were added to the first-year medical student curriculum in the 2019-2020 year, and five more were added to the first and second-year medical student curriculum in the 2020-2021 year.

One example is a small group CBL session focused on a patient who presented with acute nephrolithiasis. The LO included during that week was “apply principles of Choosing Wisely when considering diagnostic testing (focus on acute presentation of kidney stone management).” While the understanding of diagnosis and management is crucial to the health of the patient, students were pushed to discuss how this can be achieved through cost-effective tests and treatments (e.g., incorporating ultrasound into the diagnostic work-up). The goal of these discussions was to provide perspective on the critical role physician decision-making plays in reducing healthcare waste.

Instruments for assessment

**Figure 1:**
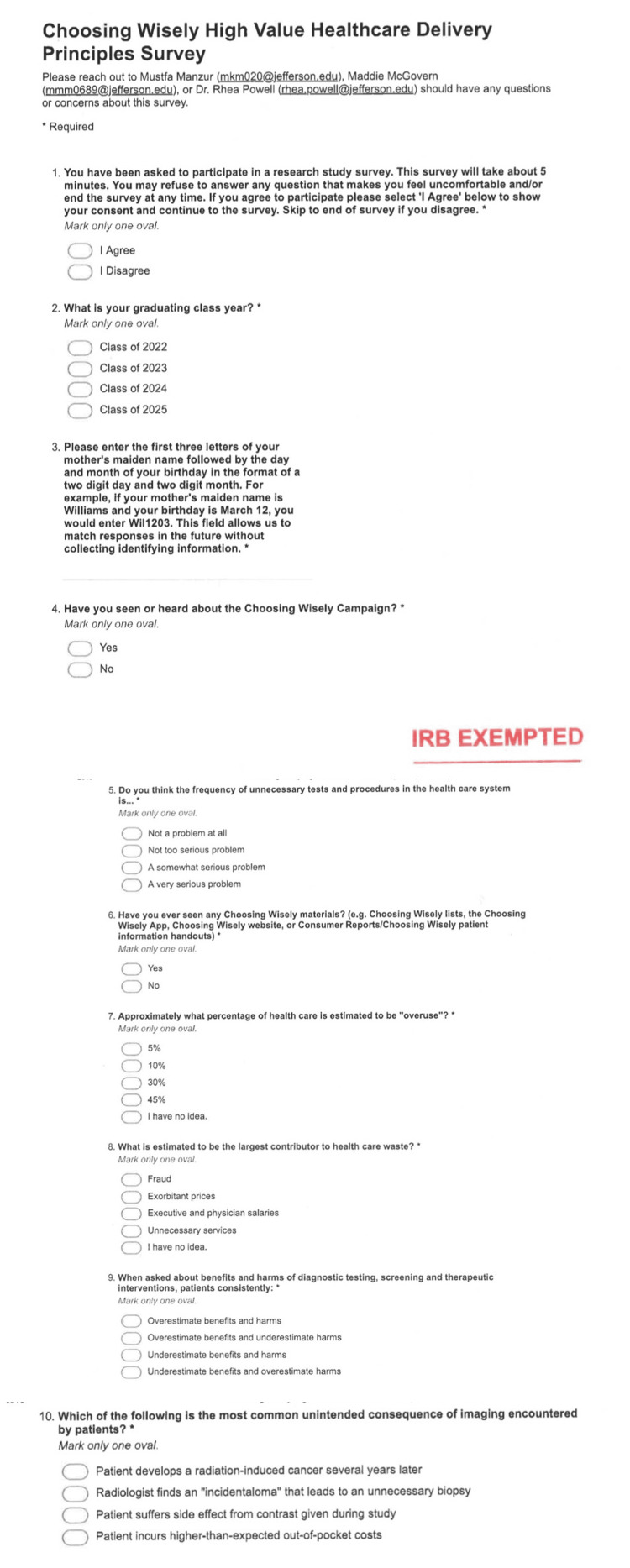
STARS Choosing Wisely Survey Questions Used During the Years 2019-2024 at Sidney Kimmel Medical College at Thomas Jefferson University

To proactively assess the impact of the intervention on (1) student awareness of the campaign and (2) student knowledge of VBC principles taught by the campaign, STARS faculty and students designed a survey in 2019 that was distributed annually to all medical students (N=1100) during July to August of the 2019-2020 to 2022-2023 academic years (n=4) (Figure 1 of Appendix). This survey was reviewed by the Thomas Jefferson University Institutional Review Board (IRB) for the involvement of human subjects (#19E.572). The survey tool was modified from a similar tool pioneered by the Dell Medical School at the University of Texas at Austin to assess the impact of curricular interventions.

The survey included two questions to assess student awareness of the campaign and four questions to assess student knowledge related to content taught via the Choosing Wisely LOs. The survey was administered annually to students to monitor the progressive efficacy of the intervention. Surveys from some years had respondents from greater than four class years due to students taking leaves of absence for various reasons; data for these students were assigned to their matriculation class. Because each survey was administered during July-August, students received varying amounts of Choosing Wisely education via LOs according to the year(s) they completed the survey. Depending on a student’s year at our medical school, the maximum number of LOs they had received at the time of annual survey completion were as follows: 2019 (N=0), 2020 (N=3), 2021 (N=8), and 2022 (N=8).

An additional instrument of competency determination employed in the study included a formal assessment of Choosing Wisely VBC principles. Students' understanding of Choosing Wisely VBC principles was assessed through items in weekly quizzes that were linked to the corresponding LOs. In total, 10 weekly quizzes over the course of the preclinical academic period contained one VBC question from a Choosing Wisely LO, totaling 10 questions. The class (by graduation year) with the most significant association between class year and knowledge of Choosing Wisely principles according to survey responses (see Data collection and statistical analysis section of Methods) was selected for further evaluation via assessment of their responses to each Choosing Wisely quiz question.

Data collection and statistical analysis 

Descriptive statistics were used to characterize survey respondents by graduation year for each year of the survey as well as responses to all survey questions. Data of respondents by graduation year includes a raw number of responses as well as a percentage of total responses by graduating year for each year of survey completion. Awareness of the Choosing Wisely campaign and materials, and correct responses to the four knowledge-based questions from Choosing Wisely LOs, were presented as percentage data. Pearson correlation analysis was conducted to determine the association between the progressive number of LO taught to students each year and (1) recognizing the campaign and materials and (2) answering each of the four knowledge-based questions correctly. Additionally, responses to the two awareness questions and the four knowledge-based questions were aggregated for each individual respondent to determine collective awareness and collective VBC concept knowledge, respectively. Logistic regression analysis was conducted to determine the presence of an association between a progressive number of LO taught to students each year and collective awareness of the Choosing Wisely campaign and knowledge of VBC principles taught by the campaign. For all statistical analyses, P-values were two-tailed, and significance was set at 5%.

A subsequent chi-square was conducted to determine the presence of an association between the graduating class year and collective awareness and knowledge. The class year with the highest degree of significance according to knowledge-based questions was evaluated further based on quiz question performance. Student performance on the 10 quiz questions from Choosing Wisely LOs is presented as a percent correct value with an associated discrimination index for each respective question. The inclusion of the discrimination index allows for the determination of high- and low-scoring learners and reflects the quality of the assessment question. The closer the value is to one, the better the item distinguishes learners who get a high score from those who get a low score. A discrimination index between 0.2 and 0.3 is considered acceptable, with indices exceeding 0.3 considered excellent. Pearson correlation analysis was also conducted to determine the association between the longitudinal inclusion of VBC LOs in the curriculum with quiz item performance.

## Results

Survey responses totaled 700 over a four-year period from 2019 to 2022 (Table 1), although response rates varied year over year from 4.3% in 2019 (N=47/1100) to 35.6% in 2020 (N=392/1100) of the medical school student population (N=1100).

**Table 1:** Survey Respondents by Graduation Year

Class Year	2019 Survey N (%)	2020 Survey N (%)	2021 Survey N (%)	2022 Survey N (%)
2021	-	50 (12.8)	7 (5.2)	-
2022	26 (55.3)	84 (21.4)	17 (12.6)	-
2023	21 (44.7)	75 (19.1)	17 (12.6)	10 (7.9)
2024	-	182 (46.4)	38 (28.1)	16 (12.7)
2025	-	1 (0.3)	56 (41.5)	61 (48.4)
2026	-	-	-	39 (31.0)
Total	47	392	135	126

Collective awareness of the Choosing Wisely campaign (Table 2) increased year over year from 2019 (39.4%) to 2022 (91.7%). The percentage of students that had seen campaign materials (“Seen Campaign Materials?”) or heard of the Choosing Wisely campaign (“Heard of Choosing Wisely?”) increased from 36.2% to 84.9% and 42.6% to 98.4% from 2019 to 2022, respectively.

**Table 2:** Awareness of Campaign and Knowledge of Curriculum Content by Percentage

Question	2019 Survey (%)	2020 Survey (%)	2021 Survey (%)	2022 Survey (%)
Collective Awareness	39.4	45.4	88.5	91.7
Heard of Choosing Wisely?	42.6	51.8	77.0	98.4
Seen Campaign Materials?	36.2	39.0	100.0	84.9
Collective VBC Concept Knowledge	64.4	67.8	74.3	74.4
30% of Care May Be Wasteful	44.7	49.5	64.4	61.1
Tests Are Main Waste Contributor	78.7	74.7	88.1	83.3
Overestimation of Test Benefits	74.5	82.1	83.7	81.7
Imaging Knowledge Question	59.6	64.8	60.7	71.4

Collective VBC concept knowledge (Table 2) also increased year over year from 2019 (64.4%) to 2022 (74.4%). The percentage of students that correctly identified 30% of health care is estimated to be wasteful (“30% of Care May Be Wasteful”) was higher in 2021 (64.4%) and 2022 (61.1%) compared to 2019 (44.7%) and 2020 (49.5%). The percentage of students that correctly identified “Unnecessary Tests and Services” as the largest contributor to health care waste (“Tests Are Main Waste Contributor”) increased in a similar fashion, with 88.1% in 2021 and 83.3% in 2022 compared to 78.7% in 2019 and 74.7% in 2020. The percentage of students that correctly identified patients consistently “Overestimate Benefits and Underestimate Harms” of diagnostic testing, screening, and therapeutic interventions (“Overestimation of Test Benefits”) increased from 2019 (74.5%) to 2020 (82.1%) and then remained consistent in 2021 (83.7%) and 2022 (81.7%). Finally, the percentage of students who correctly identified the question related to unintended consequences of imaging (“Imaging Knowledge Question”) increased from 59.6% in 2019 to 71.4% in 2022.

There was a statistically significant positive correlation between increased exposure to Choosing Wisely campaign LOs collective awareness of the campaign (0.828, p<0.0001) and collective VBC concept knowledge (0.934, p<0.001) (Table 3). There was an additional significant positive correlation between increased exposure to LOs and the percentage of respondents that correctly identified that 30% of health care is estimated to be wasteful (0.984, p=0.016).

**Table 3:** Association Between Increased LOs and Awareness or Knowledge* *Coefficients and p-values bolded were statistically significant, defined as p<0.05. LOs, learning objectives

Question	Correlation Coefficient	P-Value
Collective Awareness	0.828	<0.0001
Heard of Choosing Wisely?	0.927	0.073
Seen Campaign Materials?	0.944	0.056
Collective VBC Concept Knowledge	0.934	<0.001
30% of Care May Be Wasteful	0.984	0.016
Tests Are Main Waste Contributor	0.766	0.234
Overestimation of Test Benefits	0.825	0.175
Imaging Knowledge Question	0.519	0.481

Chi-square was employed to determine the association between class year and progressive correct responses to all survey questions assessing knowledge of VBC concepts (Table 4). All classes had significant associations between class year and awareness of the campaign. The class of 2024 had the most significant association between class year and knowledge of VBC principles (p<0.001).

**Table 4:** Association Between Class Year and Collective Awareness or Collective Knowledge* *P-values highlighted in bolded were statistically significant, defined as p<0.05.

Class	Collective Awareness (P-Value)	Collective Knowledge (P-Value)
2021	0.005	0.183
2022	0.005	0.253
2023	0.005	0.072
2024	0.005	<0.001
2025	0.004	0.002
2026	0.005	0.933

A focused analysis was conducted on the class of 2024’s quiz performance with regard to items testing VBC knowledge over the preclinical years of medical school (years 1 and 2). Table 5 displays the content of each question over the course of the preclinical curriculum’s organ-system-based blocks, along with the respective performance for each item. For each item, the percentage of students answering the item correctly is reported, along with its discrimination index. The mean percentage correct was 81.5%, with a mean discrimination index of 0.18.

**Table 5:** Class of 2024 Quiz Performance on Principles of VBC* *Indices bolded were considered acceptable, defined as >0.20. VBC, value-based care

Curriculum Block (Question Focus)	Correct Response (%)	Discrimination Index
Block 1 (Choosing Wisely)	84%	0.28
Block 2 (Cost of Care*)	63%	0.35
Block 3A (VBC)	47%	0.28
Block 3B (Choosing Wisely)	92%	0.08
Block 5 (VBC)	75%	0.25
Block 5 (VBC)	99%	0.01
Block 6 (Choosing Wisely)	100%	0.0
Block 6 (Cost of Care*)	72%	0.34
Block 6 (Choosing Wisely)	84%	0.16
Block 6 (Outcome Measures)	99%	0.01

There was a non-significant positive correlation observed between the longitudinal inclusion of VBC LOs in the curriculum with quiz item performance. The value of R was 0.4783 (p=0.162).

## Discussion

This study assessed the impact of incorporating VBC principles into undergraduate medical school curricula at an allopathic medical school in the northeastern United States. Seven hundred total responses were obtained from sequential class years of medical students over a four-year longitudinal period, providing a large sample size for analysis. In addition to knowledge, an appreciation for the importance of VBC and healthcare costs is the very first step in tackling this problem. The Choosing Wisely campaign has achieved a wider recognition of the harms of unnecessary care generally within the medical community and within this student body, less than 40% had seen materials related to the campaign in 2019 compared to 85% in 2022 [6-13].

This increased awareness also translated to a greater understanding of the magnitude of the problem low-value care and medical waste posed to medicine. The ​​percentage of students who believed the high frequency of unnecessary medical tests and procedures was a very serious problem increased year over year for four years straight (23.4% in 2019 to 38.9% in 2022), with a significant positive correlation between increased exposure to Choosing Wisely campaign LOs collective awareness of the campaign (p<0.0001). Although collective awareness of the campaign progressively increased year over year, there was a moderate decline in awareness of campaign materials and knowledge of several specific VBC principles from 2021 to 2022. This may be attributed to an extension of the negative effects of the COVID-19 pandemic on medical student education, which is significant [14,15].

We also hypothesized that students would improve their knowledge of VBC principles as a result of the study intervention. This was primarily undertaken by incorporating Choosing Wisely recommendations into small group CBL sessions as LO in the medical school’s first- and second-year curriculum. This teaching format was chosen to give students the opportunity to engage in discussions and further develop their clinical reasoning capacity. CBL has been demonstrated to be effective in enhancing complex cognitive functions as it relates to medical decision-making and can also improve patient outcomes [16]. Multiple studies report that medical students and physicians have successfully used CBL to attain the necessary medical knowledge for hepatitis C virus treatment, diabetes treatment in primary care, chlamydia screening, and adverse drug reactions, leading to improved patient outcomes in clinical practice [17-20].

Increased LOs resulted in a significant positive association with collective awareness of the Choosing Wisely campaign (p<0.0001) and knowledge of VBC concepts (p<0.001) among all survey respondents. Moreover, increased LO exposure was significantly associated with correctly identifying that 30% of health care is estimated to be wasteful (p=0.016). Additional investigation of the association between class year and collective knowledge of VBC concepts was significant in the classes of 2024 (p<0.001) and 2025 (p=0.002). These classes had the greatest longitudinal exposure to VBC LOs, beginning in their first year of medical school. These results reinforce the effectiveness of enhancing discussion and clinical knowledge via increased utilization of VBC-focused LOs in small-group CBL environments.

The longitudinal addition of LOs in the preclinical curriculum was also found to enhance students’ self-directed learning and positively influence performance on quiz items relating to VBC. The quiz performance of the class of 2024 was investigated further due to (1) the high number of survey respondents from this class and (2) the significant association between class year and collective knowledge of VBC principles as determined by our study. Overall, test items focused on VBC had an average score of 81.5% with a discrimination index of 0.18, with an index >0.20 on five of 10 questions. A non-significant, positive correlation between increased LOs and improved quiz performance was observed. The lack of statistical significance may be attributable to a limited sample size, as only 10 items were analyzed for one student cohort. Additionally, quiz performance has been shown to successfully predict formative medical student knowledge in the future, which helps to support the validity of these findings with regard to knowledge of VBC principles over time [21].

Studies of educational interventions related to Choosing Wisely principles have traditionally focused on healthcare professionals who have completed their training, such as board-certified physicians and nurses [22-25]. However, this study aims to explore the effectiveness of intervening during UME. Student-led initiatives, with proper institutional and faculty support, are simple yet effective interventions that promote prioritization of value-based earlier in the medical training process. Additionally, student involvement in medical school curricular change has been shown to increase awareness of curricular initiatives and interest in academic medicine as a future career [26,27]. This model could serve as an example to encourage other medical schools in the United States and abroad to include students in discussions regarding curricular changes, empowering them to advocate for changes they believe are worthwhile. This may also allow for greater synergy between the medical school curriculum and student-led initiatives outside the classroom.

Limitations

Several factors limit this study. First, the data collection was confined to a single undergraduate medical institution, which might restrict the generalizability of the findings to a broader population. The reliance on survey data introduces potential biases, notably recall bias and response bias, inherent in self-reported information. The voluntary nature of the survey led to low response rates, with varying participation levels among different cohorts across the years. Although the surveys were consistently distributed during the fall semester, the disparities in response rates among cohorts and varying distribution dates might have introduced fluctuations in the data.

The education received from LOs varied depending on the year of matriculation or completion of the study, potentially impacting the consistency and depth of exposure to VBC principles. The six knowledge-based survey questions also may not adequately assess a comprehensive understanding of these principles. Moreover, the study did not account for external factors influencing participants' exposure to VBC principles through other curricular or extracurricular activities, which might have augmented knowledge and shaped attitudes. Therefore, observed enhancements in attitudes and knowledge surrounding Choosing Wisely could be influenced by diverse factors beyond the specific interventions outlined in this study.

Future studies should strive for a more diverse and extensive sample by including students from various institutions. Additionally, enhancement of the survey tool and expansion of the duration of data collection (by following students into their professional careers, for example) could offer a more comprehensive evaluation of VBC interventions. However, conducting such a longitudinal study presents logistical challenges. It is also important to consider external factors that might influence clinical practice beyond educational interventions, such as healthcare policy changes and technological advancements.

## Conclusions

Implementation of curricular content related to VBC principles from the Choosing Wisely campaign during small group CBL sessions significantly increased medical student awareness of the campaign and knowledge of VBC concepts. This study underscores the power of integrating student advocacy in curriculum development to instill early VBC education. The success of this approach emphasizes the need for broader implementation, showcasing the pivotal role of VBC in shaping the future of global healthcare.
